# Torsion of Appendiceal Mucinous Neoplasm: A Rare Cause of Right Lower Quadrant Abdominal Pain

**DOI:** 10.7759/cureus.95246

**Published:** 2025-10-23

**Authors:** Alexander F Bowers, David M Dodson, Alexander J Monson, Courtney Thomas, Aashish Rajesh

**Affiliations:** 1 Surgery, University of Texas (UT) Health San Antonio, San Antonio, USA; 2 Pathology, University of Texas (UT) Health San Antonio, San Antonio, USA

**Keywords:** appendectomy, appendiceal mucinous neoplasm, appendiceal mucocele, appendiceal torsion, pseudomyxoma peritonei

## Abstract

Appendiceal mucinous neoplasms are rare, and torsion of the appendix is exceptionally uncommon. Rupture of an appendiceal mucinous neoplasm can lead to pseudomyxoma peritonei, a condition with high morbidity. We present the case of a 64-year-old male patient with five days of right lower quadrant abdominal pain. Cross-sectional imaging of the abdomen and pelvis revealed a five-centimeter-long, tubular, ring-enhancing lesion, concerning for a mucocele. Intraoperatively, a 360-degree appendiceal torsion was identified, prompting conversion from laparoscopy to laparotomy to avoid mucocele rupture. Pathology confirmed a low-grade appendiceal mucinous neoplasm with negative margins. The patient recovered uneventfully and remained asymptomatic at follow-up. This case underscores the deceptive presentation of a torsed appendiceal mucocele and highlights important technical considerations for optimal operative management of this pathology.

## Introduction

The first case of appendiceal mucocele was described by Rokitansky in 1842, and appendiceal carcinoma was reported several decades later in 1882 [1,2]. In 1918, Payne recorded the first case of torsion of the appendix, a rare phenomenon that may occur either primarily or secondary to underlying pathology [3]. Despite over a century of recognition, these conditions remain uncommon and may be overlooked as causes of abdominal pain [4].

Appendiceal mucoceles account for only 0.2% to 0.3% of appendectomy specimens. Their clinical presentation often mimics acute appendicitis, making preoperative differentiation challenging [4-6]. Mucocele formation is frequently secondary to mucinous appendiceal neoplasms, which are classified as low-grade appendiceal mucinous neoplasm (LAMN), high-grade mucinous adenocarcinoma, and signet-ring cell carcinoma [7].

Although LAMN is the least invasive of the three types of mucinous appendiceal neoplasms, all three pathologies can result in mucocele formation. The most feared complication is pseudomyxoma peritonei (PMP), which arises from mucocele rupture with peritoneal seeding of neoplastic mucinous cells. PMP carries significant morbidity, often requiring cytoreductive surgery and intraperitoneal chemotherapy [8].

The coexistence of torsion with a LAMN-associated mucocele is exceptionally rare [6]. Awareness of this entity is important, as intraoperative findings may necessitate modification of surgical strategy to prevent rupture and subsequent PMP. We present a case of LAMN diagnosed with preoperative imaging and intraoperatively found to have appendiceal torsion, which was managed successfully with an appendectomy without rupture.

## Case presentation

A 64-year-old male presented to the emergency department with a five-day history of right lower quadrant (RLQ) abdominal pain. He denied fevers, chills, nausea, or vomiting. His past surgical history included prostatectomy for benign prostatic hyperplasia. He was hemodynamically normal with a normal white blood cell count (7.84 × 10³ per microliter). Examination revealed localized tenderness in the right lower quadrant without signs of peritonitis.

**Figure 1:**
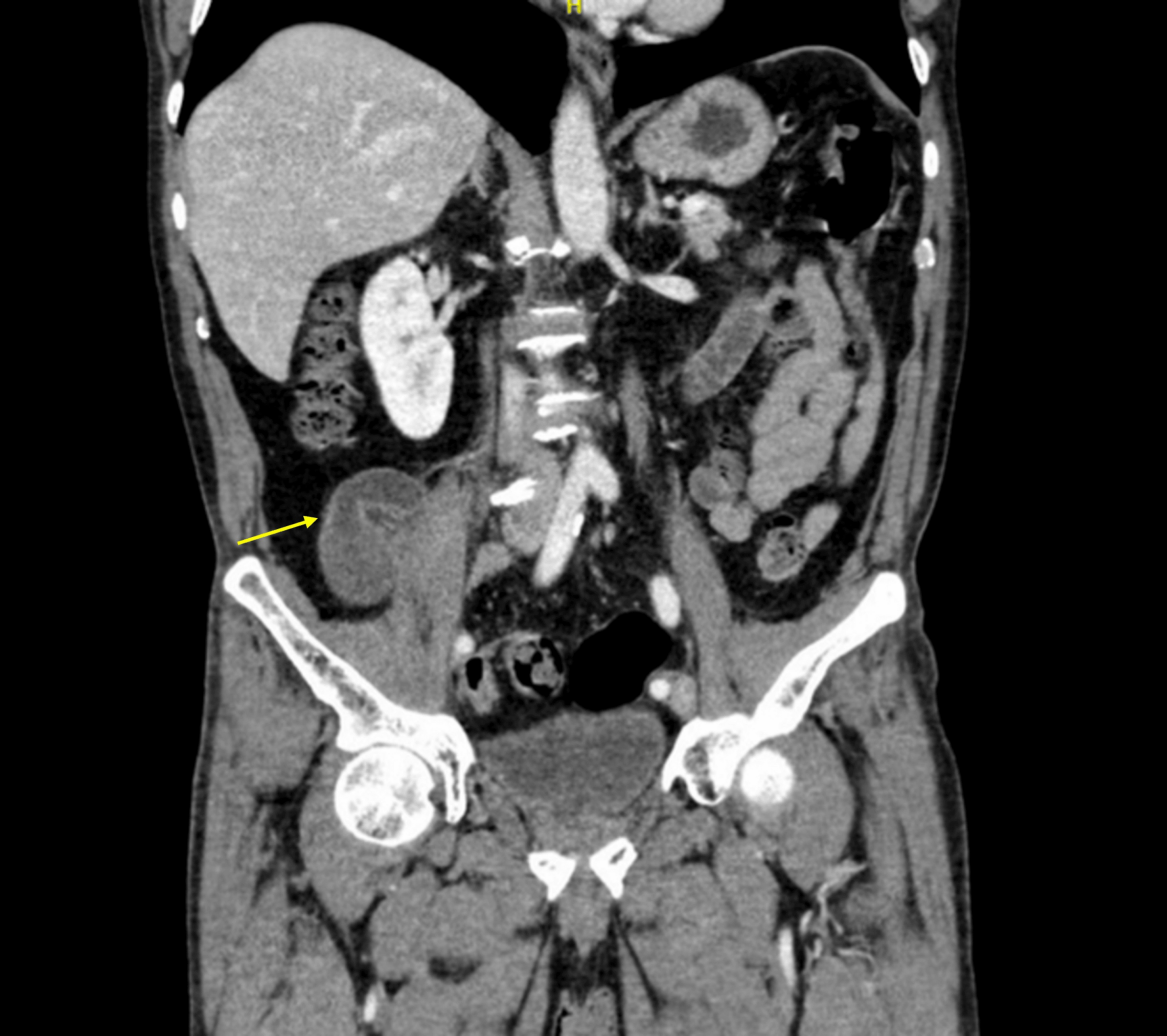
Coronal section of computed tomography imaging showing distended appendix (arrow).

**Figure 2:**
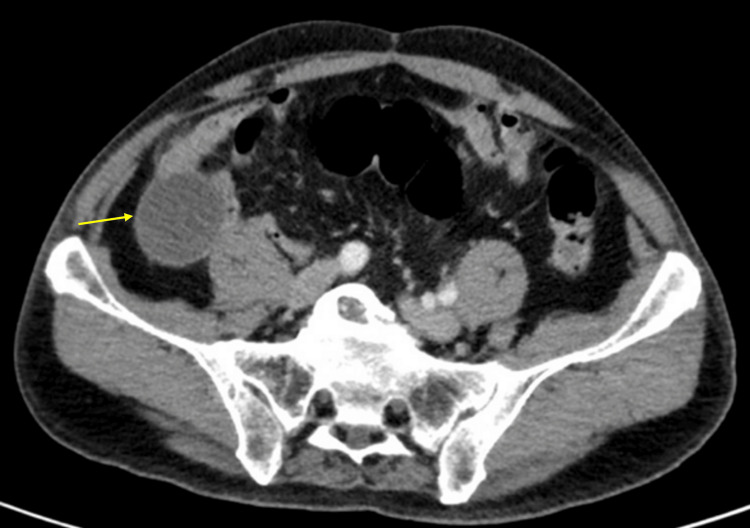
Axial section of computed tomography imaging showing appendiceal mucocele (arrow).

Contrast-enhanced computed tomography (CT) of the abdomen and pelvis demonstrated a well-defined, tubular, ring-enhancing lesion in the RLQ measuring five centimeters, consistent with an appendiceal mucocele (Figures 1, 2).

**Figure 3:**
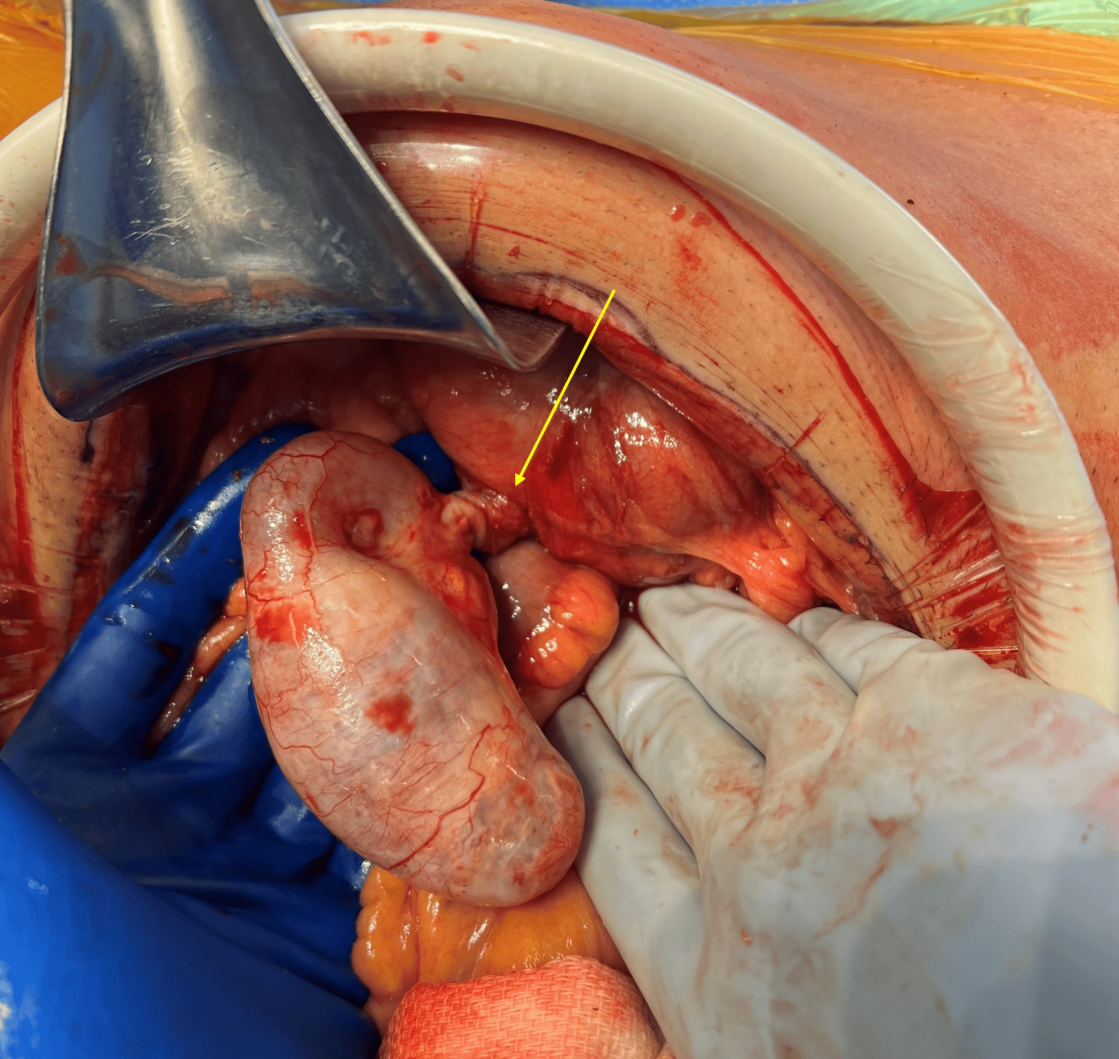
Appendiceal mucocele with 360 degrees of torsion noted around the base (arrow).

The patient was started on intravenous fluids and antibiotics and consented for surgery. Diagnostic laparoscopy revealed a distended appendix with 360 degrees of torsion around its base and a distended mucus-filled body. Given the high risk of rupture and peritoneal seeding with laparoscopic handling of the tensely distended appendix, the procedure was converted to open appendectomy through a lower midline laparotomy (Figure 3).

**Figure 4:**
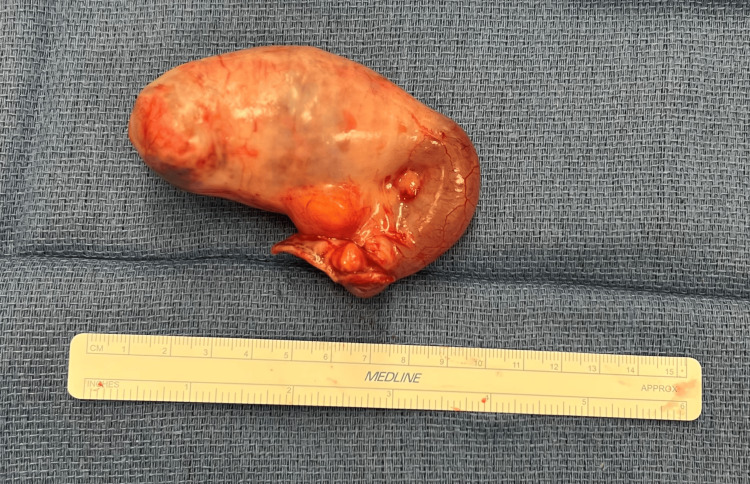
Operative specimen showing appendix with mucocele.

The appendix was gently elevated from the pelvis, detorsed, and transected with a linear stapler along with a healthy cecal cuff (Figure 4).

Postoperative histopathology confirmed a LAMN with mucocele, staged as pTis with negative margins and no metastatic disease. The patient’s recovery was uneventful, and he was discharged home tolerating a regular diet. At two-week follow-up, he remained asymptomatic. He was referred to medical oncology for longitudinal care, and was scheduled for a diagnostic colonoscopy in three months and CT imaging surveillance in one year.

## Discussion

Torsion of an appendiceal mucocele is exceedingly rare, with limited literature to guide clinical decision-making [1]. Appendiceal mucocele itself is uncommon and encompasses a spectrum of etiologies from benign cysts to malignant neoplasms such as LAMN [5]. Malignant mucinous neoplasms are particularly concerning due to the risk of PMP if rupture occurs [9].

PMP is characterized by intraperitoneal accumulation of mucin and neoplastic epithelial cells, leading to progressive mucinous ascites, bowel obstruction, and potential pleural involvement. Management requires cytoreductive surgery and hyperthermic intraperitoneal chemotherapy, but recurrence rates remain high, underscoring the importance of preventing rupture [10,11].

Clinical presentation of appendiceal mucinous neoplasms can mimic acute appendicitis when torsion occurs. Preoperative imaging with CT typically reveals a cystic, dilated, tubular structure in the RLQ of the abdomen, but distinguishing benign from malignant mucoceles and detecting torsion preoperatively is difficult [12,13]. In our case, CT identified a ring-enhancing lesion consistent with a mucocele, though torsion was only appreciated intraoperatively. Laparoscopic exploration was initially undertaken; however, the degree of torsion and risk of rupture prompted conversion to open laparotomy. Historically, open resection has been advocated for mucoceles to minimize manipulation and reduce the risk of PMP. Although some authors report successful laparoscopic management, limited follow-up raises concerns regarding occult rupture and delayed recurrence [14-16]. Recurrence of LAMN has been documented up to 10 years post-resection [17,18]. In the era of advanced laparoscopic and robotic surgery, extreme caution is imperative when proceeding with minimally invasive approaches for appendiceal mucoceles to prioritize safe oncologic removal without rupture.

Complete resection with negative margins is critical for favorable prognosis in LAMN [16]. Surveillance following appendectomy remains debated. The American Society of Colon and Rectal Surgeons recommends two years of monitoring with tumor markers and cross-sectional imaging, whereas the Chicago Consensus Working Group advises no surveillance in cases with negative margins and no perforation or extra-appendiceal mucin [19]. These discrepancies highlight the need for further research on optimal follow-up protocols.

## Conclusions

Appendiceal mucocele with torsion is a rare clinical entity that can present as right lower quadrant abdominal pain. Rupture of mucinous neoplasms may lead to pseudomyxoma peritonei, and surgical strategies must be guided by intraoperative findings and cautious handling. In high-risk cases, open approach may be appropriate to minimize the risk of rupture. Ongoing research is needed to refine long-term surveillance guidelines for LAMNs.
